# Syntheses and structures of piperazin-1-ium *A*Br_2_ (*A* = Cs or Rb): hybrid solids containing ‘curtain wall’ layers of face- and edge-sharing *A*Br_6_ trigonal prisms

**DOI:** 10.1107/S2056989019010375

**Published:** 2019-07-26

**Authors:** Kirstie A. Rickaby, Alexandra M. Z. Slawin, William T. A. Harrison

**Affiliations:** a Department of Chemistry, University of Aberdeen, Meston Walk, Aberdeen AB24 3UE, Scotland; b Department of Chemistry, University of St Andrews, St Andrews KY16 9ST, Scotland

**Keywords:** crystal structure, hybrid solid, caesium, rubidium, trigonal prism

## Abstract

The isostructural title compounds, poly[piperazin-1-ium [di-μ-bromido-caesium]], {(C_4_H_11_N_2_)[CsBr_2_]}_*n*_, and poly[piperazin-1-ium [di-μ-bromido-rubidium]], {(C_4_H_11_N_2_)[RbBr_2_]}_*n*_, contain singly-protonated piperazin-1-ium cations and unusual *A*Br_6_ (*A* = Cs or Rb) trigonal prisms. The prisms are linked into a distinctive ‘curtain wall’ arrangement propagating in the (010) plane by face and edge sharing. In each case, a network of N—H⋯N, N—H⋯Br and N—H⋯(Br,Br) hydrogen bonds consolidates the structure.

## Abstract 2

The isostructural title compounds feature striking layers of *A*Br_6_ (*A* = Cs, Rb) trigonal prisms sharing faces and edges.

## Chemical context   

Oxide perovskites of generic formula *AB*O_3_, where *A* and *B* are metal ions, have been studied for decades because of their physical properties and structural variety (Tilley, 2016 ▸). The aristotype (highest-possible symmetry) for this familiar structure type is a cubic network (space group *Pm* 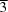 *m*) of vertex-sharing, regular, BO_6_ octa­hedra encapsulating the *A* cations in 12-coordinate cavities bounded by eight octa­hedra, but lower symmetry structures are very common (Woodward, 1997 ▸). More recently, ‘hybrid’ *RMX*_3_ perovskites containing organic cations and *MX*_3_ (*M* = Pb, Sn…; *X* = halide ion) octa­hedral networks have attracted intense inter­est because of their remarkable photophysical properties (Xu *et al.*, 2019 ▸; Stylianakis *et al.*, 2019 ▸; Zuo *et al.*, 2019 ▸). A number of different organic cations occur in these hybrid structures, one of which is the doubly protonated C_4_H_12_N_2_^2+^ piperizinium (or piperazin-1,4-diium) ion as found in the C_4_H_12_N_2_·*A*Cl_3_·H_2_O (*A* = K, Rb, Cs) family (Paton & Harrison, 2010 ▸) and C_4_H_12_N_2_·NaI_3_ (Chen *et al.*, 2018 ▸).
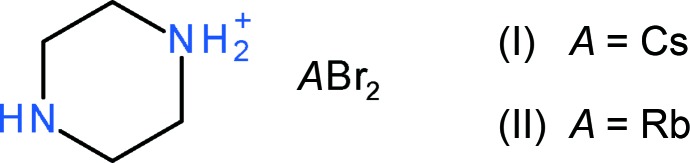


As an extension of these studies, we now describe the title hybrid compounds, containing the singly protonated C_4_H_11_N_2_^+^ piperazin-1-ium cation, which have a generic formula of *RMX*_2_ and totally different crystal structures to *RMX*_3_ hybrid perovskites.

## Structural commentary   

Compounds (I) and (II) are isostructural and crystallize in the ortho­rhom­bic space group *Pbcm*. The smaller unit-cell volume (by 5.3%) of (II) presumably reflects the smaller ionic radius (Shannon, 1976 ▸) of the Rb^+^ cation (*r* = 1.66 Å) compared to Cs^+^ (*r* = 1.81 Å). This structure description will focus on (I) and note significant differences for (II) where applicable.

**Figure 1:**
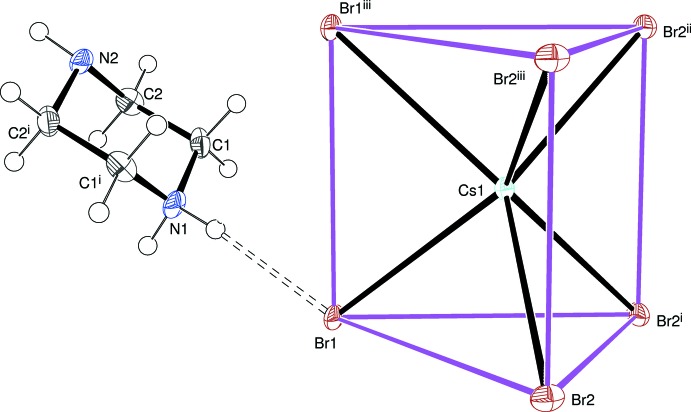
The asymmetric unit of (I) showing 50% displacement ellipsoids expanded to show the complete organic cation and the caesium coordination polyhedron. The N—H⋯Br hydrogen bond is shown as a double-dashed line. The purple lines linking the bromine atoms emphasize the trigonal–prismatic shape of the CsBr_6_ polyhedron. Symmetry codes: (i) *x*, *y*, 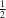 − *z*; (ii) *x* − 1, *y*, 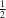 − *z*; (iii) *x* − 1, *y*, *z*.

The asymmetric unit of (I) consists of two methyl­ene groups, an NH group and an NH_2_^+^ group; both nitro­gen atoms and their attached H atoms lie on a (001) crystallographic mirror plan (at *z* = 1/4 for the asymmetric atoms). The structure is completed by a caesium atom [site symmetry *m*(001), Wyckoff site 4*d*] and two bromine atoms: Br1 [*m*(001); 4*d*] and Br2 (2[100]; 4*c*). The structure of (I) is shown in (Fig. 1 ▸).

The complete C_4_H_11_N_2_^+^ cation is generated by reflection to result in a typical (Brüning *et al.*, 2009 ▸) chair conformation for the ring: N1 and N2 deviate from the mean plane of C1/C2/C1^i^/C2^i^ [symmetry code: (i) *x*, *y*, 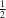 − *z*] by 0.656 (5) and −0.682 (4) Å, respectively. The H atom of the neutral N2—H3*N* group has an equatorial orientation with respect to the ring.

The caesium coordination polyhedron in (I) is completed by crystal symmetry, resulting in a distinctive CsBr_6_ trigonal prism (Fig. 1 ▸): the prism has longitudinal (001) mirror symmetry, with the Br1 atoms and the metal atom lying on the mirror. The mean Cs—Br bond length based on four distinct Cs—Br bonds (Table 1 ▸) is 3.573 Å [mean Rb—Br bond length for (II) = 3.461 Å; Table 2 ▸]. These data may be compared with the shortest Cs—Br separation of 3.716 Å in CsBr (8-coord­inate caesium chloride structure) and the shortest Rb—Br separation of 3.427 Å in RbBr (6-coordinate rocksalt structure).

In (I), the prism ends (Br1/Br2/Br2^i^ and Br1^iii^/Br2^ii^/Br2^iii^; see Fig. 1 ▸ for symmetry codes) are parallel by symmetry and separated by 4.5787 (8) Å, *i.e*., the *a* unit-cell parameter, hence there is no twisting of the end faces and the Br⋯Br⋯Br angles vary from 56.65 (1)–61.68 (1)° [the equivalent prism-end separation for (II) is 4.4675 (13) Å]. The caesium cation in (I) is not quite equidistant from the prism-ends mentioned in the previous sentence, being displaced from them by 2.3177 (6) and 2.2605 (5) Å, respectively. The equivalent data for the Rb atom in (II) are 2.2581 (9) and 2.2091 (9) Å, respectively. The bond-valence sum (BVS) for Cs1 (in valence units) using the formalism of Brese & O’Keeffe (1991 ▸) in (I) is 1.12 and the equivalent value for Rb1 in (II) is 0.95 (expected value in both cases = 1.00). This indicates that the bond valences of these cations are satisfied without notable underbonding or overbonding in these unusual coordination environments.

It may be finally noted that the bromide ions have very different coordination environments: Br1 bridges to two metal atoms [Cs1—Br1—Cs1^iv^ = 78.17 (2) in (I); Rb1—Br1—Rb1^iv^ = 79.21 (3)° in (II); symmetry code: (iv) *x* + 1, *y*, *z*] whereas Br2 has an unusual distorted square planar BrCs_4_ arrangement: the cis Cs—Br2—Cs bond angles in (I) vary between 80.367 (13) and 100.865 (16)°; the five atoms are exactly co-planar by symmetry.

## Supra­molecular features   

**Figure 2:**
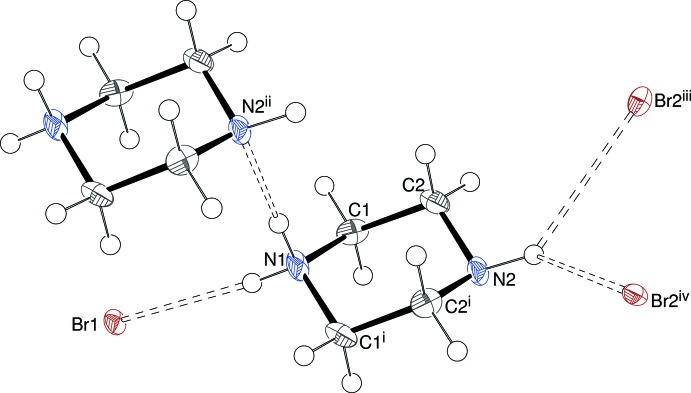
Detail of the structure of (I) showing the hydrogen-bonding environment of the C_4_H_11_N_2_^+^ cation; symmetry codes: (i) *x*, *y*, 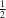 − *z*; (ii) *x* + 1, *y*, *z*; (iii) 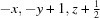; (iv) 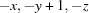.

The extended structure of (I) is consolidated by hydrogen bonds (Fig. 2 ▸, Table 3 ▸. The N1—H1*N*⋯N2 bond from the protonated NH_2_^+^ group to the unprotonated N atom in an adjacent mol­ecule links the organic cations into [100] chains with adjacent cations related by translation symmetry and the N1—H2*N*⋯Br1 bond connects the organic cation to the inorganic network. The neutral N2—H3*N* moiety forms a bifurcated N—H⋯(Br2,Br2) hydrogen bond; the H⋯Br contacts are long at 3.07 (3) Å but given their apparent role in bridging the (010) CsBr_2_ layers we judge them to be structurally significant. The hydrogen-bonding scheme for (II) (Table 4 ▸) is almost identical to that in (I).

**Figure 3:**
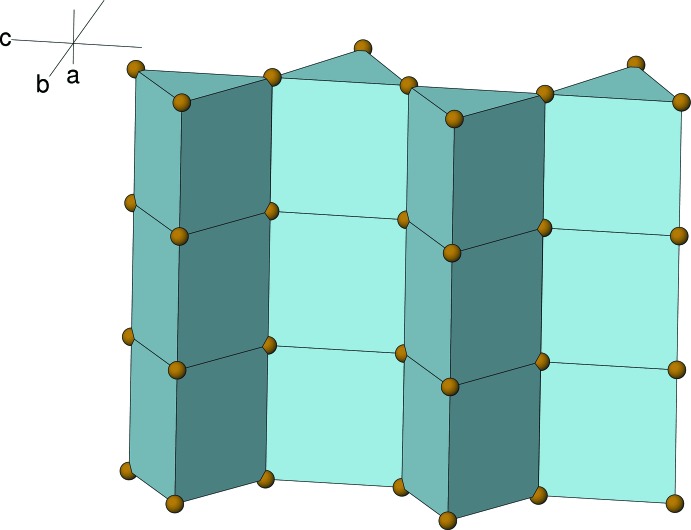
Polyhedral view of part of an (010) layer of CsBr_6_ trigonal prisms in (I).

**Figure 4:**
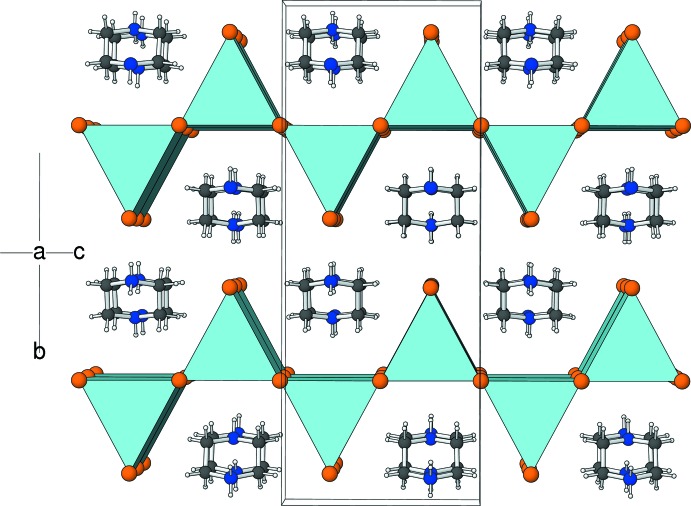
The unit-cell packing in (I) viewed down [100]. Note the ‘saw-tooth’ arrangement of stacks of CsBr_6_ prisms with respect to the [001] direction.

The CsBr_6_ prisms in (I) are linked into a striking (010) ‘curtain wall’ arrangement (Fig. 3 ▸) by face sharing in the [100] direction and edge sharing (*via* a pair of Br2 atoms) in the [001] direction; the Cs⋯Cs separation through the prism-ends is 4.5787 (8) Å (by the symmetry operations *x* + 1, *y*, *z* and *x* − 1, *y*, *z*) and the separation between metal ions in adjacent columns is 5.42014 (12) Å (symmetry operations *x*, 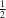 − *y*, −*z* and *x*, 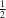 − *y*, 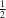 + *z*). The equivalent data for the Rb atoms in (II) are 4.4675 (13) and 5.2338 (14) Å, respectively. When viewed down [100], the prisms adopt a ‘saw-tooth’ arrangement with respect to the [010] direction, with alternating columns of prisms pointing ‘up’ and ‘down’ (Fig. 4 ▸).

## Database survey   

So far as we are aware, the *RA*Br_2_ topology of the title compounds is a novel one. A search of the Cambridge Structural Database (CSD, version 5.40, last update 19 May 2019; Groom *et al.*, 2016 ▸) for the mono-protonated C_4_H_11_N_2_^+^ cation returned 55 crystal structures but none of them bear a close resemblance to the title compound. As noted in the chemical context section, the doubly protonated C_4_H_12_N_2_^2+^ species occurs in several hybrid *RMX*_3_ perovskites including C_4_H_12_N_2_·*A*Cl_3_·H_2_O with *A* = K (CSD refcode GUYMIX), Rb (GUYMOD) and Cs (GUYMUJ) (Paton & Harrison, 2010 ▸) and C_4_H_12_N_2_·NaI_3_ (MEXMAG; Chen *et al.*, 2018 ▸).

## Synthesis and crystallization   

Compound (I) was prepared by adding 0.213 g (1.0 mmol) of CsBr and 0.086 g (1.0 mmol) of piperazine to 11.0 ml (1.1 mmol) of a 0.1 *M* HBr solution in a Petri dish to result in a clear solution. Colourless rods of (I) formed after a few days as the water evaporated. Colourless rods of (II) were prepared in the same way, with 0.165 g (1.0 mmol) of RbBr replacing the CsBr. The qu­antity of acid appears to be critical to the syntheses of (I) and (II): smaller amounts lead to recrystallized CsBr and RbBr and larger amounts lead to different structures containing doubly protonated C_4_H_12_N_2_^2+^ cations.

## Refinement   

Crystal data, data collection and structure refinement details are summarized in Table 5 ▸. The N-bonded H atoms were located in difference-Fourier maps: for (I), their positions were freely refined, for (II) they were refined as riding atoms in their as-found relative positions. The C-bound H atoms were placed geometrically (C—H = 0.99 Å) and refined as riding atoms for both structures. The constraint *U*_iso_(H) = 1.2*U*_eq_(carrier) was applied in all cases. The displacement ellipsoids for the C and N atoms in (II) refined to somewhat elongated shapes suggestive of positional disorder of the C_4_H_11_N_2_^+^ cations but attempts to model this did not lead to a significant improvement in fit.

## Supplementary Material

Crystal structure: contains datablock(s) I, II, global. DOI: 10.1107/S2056989019010375/su5506sup1.cif

Structure factors: contains datablock(s) I. DOI: 10.1107/S2056989019010375/su5506Isup2.hkl

Structure factors: contains datablock(s) II. DOI: 10.1107/S2056989019010375/su5506IIsup3.hkl

CCDC references: 1941895, 1941894

Additional supporting information:  crystallographic information; 3D view; checkCIF report

## Figures and Tables

**Table 1:** Selected bond lengths (Å) for (I)

Cs1—Br2^iii^	3.5157 (5)	Cs1—Br1	3.6228 (6)
Cs1—Br2	3.5801 (5)	Cs1—Br1^iii^	3.6392 (7)

Symmetry code: (iii) 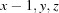.

**Table 2:** Selected bond lengths (Å) for (II)

Rb1—Br2^iii^	3.4157 (8)	Rb1—Br1	3.5013 (9)
Rb1—Br2	3.4659 (8)	Rb1—Br1^iii^	3.5068 (9)

Symmetry code: (iii) 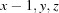.

**Table 3:** Hydrogen-bond geometry (Å, °) for (I)

*D*—H⋯*A*	*D*—H	H⋯*A*	*D*⋯*A*	*D*—H⋯*A*
N1—H1*N*⋯N2^ii^	0.92 (4)	1.95 (4)	2.868 (4)	179 (3)
N1—H2*N*⋯Br1	0.84 (4)	2.45 (4)	3.284 (3)	174 (4)
N2—H3*N*⋯Br2^iii^	0.95 (4)	3.07 (3)	3.756 (2)	130 (1)
N2—H3*N*⋯Br2^iv^	0.95 (4)	3.07 (3)	3.756 (2)	130 (1)

Symmetry codes: (ii) 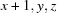; (iii) 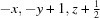; (iv) 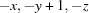.

**Table 4:** Hydrogen-bond geometry (Å, °) for (II)

*D*—H⋯*A*	*D*—H	H⋯*A*	*D*⋯*A*	*D*—H⋯*A*
N1—H2*N*⋯N2^ii^	0.91	1.92	2.825 (6)	179
N1—H1*N*⋯Br1	0.91	2.40	3.300 (4)	171
N2—H3*N*⋯Br2^iii^	0.94	3.07	3.762 (3)	131
N2—H3*N*⋯Br2^iv^	0.94	3.07	3.762 (3)	131

Symmetry codes: (ii) 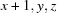; (iii) 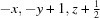; (iv) 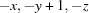.

**Table 5:** Experimental details

	(I)	(II)
Crystal data		
Chemical formula	(C_4_H_11_N_2_)[CsBr_2_]	(C_4_H_11_N_2_)[RbBr_2_]
*M*_r_	379.88	332.44
Crystal system, space group	Orthorhombic, *P* *b* *c* *m*	Orthorhombic, *P* *b* *c* *m*
Temperature (K)	93	93
*a*, *b*, *c* (Å)	4.5787 (8), 23.325 (5), 9.1828 (17)	4.4675 (13), 23.036 (7), 9.021 (3)
*V* (Å^3^)	980.7 (3)	928.4 (5)
*Z*	4	4
Radiation type	Mo *K*α	Mo *K*α
μ (mm^−1^)	11.86	13.87
Crystal size (mm)	0.20 × 0.05 × 0.05	0.20 × 0.05 × 0.05

Data collection		
Diffractometer	Rigaku Pilatus 200K CCD	Rigaku Pilatus 200K CCD
Absorption correction	Multi-scan (*CrystalClear*; Rigaku, 2013 ▸)	Multi-scan (*CrystalClear*; Rigaku, 2013 ▸)
*T*_min_, *T*_max_	0.639, 1.000	0.597, 1.000
No. of measured, independent and observed [*I* > 2σ(*I*)] reflections	11592, 959, 917	11351, 908, 771
*R*_int_	0.056	0.086
(sin θ/λ)_max_ (Å^−1^)	0.603	0.602

Refinement		
*R*[*F* ^2^ > 2σ(*F* ^2^)], *wR*(*F* ^2^), *S*	0.021, 0.055, 1.10	0.023, 0.057, 0.94
No. of reflections	959	908
No. of parameters	55	48
H-atom treatment	H atoms treated by a mixture of independent and constrained refinement	H-atom parameters constrained
Δρ_max_, Δρ_min_ (e Å^−3^)	1.34, −0.96	0.74, −0.47

Computer programs: *CrystalClear* (Rigaku, 2013 ▸), *SHELXS97* (Sheldrick, 2008 ▸), *SHELXL2018* (Sheldrick, 2015 ▸), *ORTEP-3 for Windows* (Farrugia, 2012 ▸), *ATOMS* (Shape Software, 2005 ▸) and *publCIF* (Westrip, 2010 ▸).

## supplementary crystallographic information

### Poly[piperazin-1-ium [di-µ-bromido-caesium]] (I) Crystal data

**Table:**

(C_4_H_11_N_2_)[CsBr_2_]	*D*_x_ = 2.573 Mg m^−^^3^
*M**_r_* = 379.88	Mo *K*α radiation, λ = 0.71073 Å
Orthorhombic, *P**b**c**m*	Cell parameters from 3076 reflections
*a* = 4.5787 (8) Å	θ = 2.8–27.5°
*b* = 23.325 (5) Å	µ = 11.86 mm^−^^1^
*c* = 9.1828 (17) Å	*T* = 93 K
*V* = 980.7 (3) Å^3^	Rod, colourless
*Z* = 4	0.20 × 0.05 × 0.05 mm
*F*(000) = 696	

### Poly[piperazin-1-ium [di-µ-bromido-caesium]] (I) Data collection

**Table:**

Rigaku Pilatus 200K CCD diffractometer	917 reflections with *I* > 2σ(*I*)
Radiation source: rotating anode	*R*_int_ = 0.056
ω scans	θ_max_ = 25.4°, θ_min_ = 3.5°
Absorption correction: multi-scan (CrystalClear; Rigaku, 2013)	*h* = −5→5
*T*_min_ = 0.639, *T*_max_ = 1.000	*k* = −28→28
11592 measured reflections	*l* = −11→11
959 independent reflections	

### Poly[piperazin-1-ium [di-µ-bromido-caesium]] (I) Refinement

**Table:**

Refinement on *F*^2^	Hydrogen site location: mixed
Least-squares matrix: full	H atoms treated by a mixture of independent and constrained refinement
*R*[*F*^2^ > 2σ(*F*^2^)] = 0.021	*w* = 1/[σ^2^(*F*_o_^2^) + (0.0387*P*)^2^] where *P* = (*F*_o_^2^ + 2*F*_c_^2^)/3
*wR*(*F*^2^) = 0.055	(Δ/σ)_max_ = 0.001
*S* = 1.10	Δρ_max_ = 1.34 e Å^−^^3^
959 reflections	Δρ_min_ = −0.96 e Å^−^^3^
55 parameters	Extinction correction: SHELXL2018 (Sheldrick, 2015), Fc^*^=kFc[1+0.001xFc^2^λ^3^/sin(2θ)]^-1/4^
0 restraints	Extinction coefficient: 0.0010 (2)
Primary atom site location: structure-invariant direct methods	

### Poly[piperazin-1-ium [di-µ-bromido-caesium]] (I) Special details

**Table:**

Geometry. All esds (except the esd in the dihedral angle between two l.s. planes) are estimated using the full covariance matrix. The cell esds are taken into account individually in the estimation of esds in distances, angles and torsion angles; correlations between esds in cell parameters are only used when they are defined by crystal symmetry. An approximate (isotropic) treatment of cell esds is used for estimating esds involving l.s. planes.

### Poly[piperazin-1-ium [di-µ-bromido-caesium]] (I) Fractional atomic coordinates and isotropic or equivalent isotropic displacement parameters (Å^2^)

**Table:**

	*x*	*y*	*z*	*U*_iso_*/*U*_eq_	
Cs1	0.02411 (5)	0.31176 (2)	0.250000	0.01125 (13)	
Br1	0.52127 (7)	0.43259 (2)	0.250000	0.01635 (15)	
Br2	0.53501 (7)	0.250000	0.000000	0.01654 (16)	
C1	−0.0049 (5)	0.56445 (11)	0.3834 (4)	0.0205 (7)	
H1A	0.120469	0.560779	0.470727	0.025*	
H1B	−0.158445	0.534633	0.388670	0.025*	
C2	−0.1448 (6)	0.62308 (9)	0.3811 (2)	0.0209 (5)	
H2A	−0.267978	0.628079	0.468749	0.025*	
H2B	0.008673	0.652975	0.382326	0.025*	
N1	0.1740 (7)	0.55578 (12)	0.250000	0.0217 (7)	
H1N	0.336 (9)	0.5788 (16)	0.250000	0.026*	
H2N	0.251 (8)	0.5231 (17)	0.250000	0.026*	
N2	−0.3243 (7)	0.62943 (11)	0.250000	0.0186 (6)	
H3N	−0.427 (7)	0.6650 (19)	0.250000	0.022*	

### Poly[piperazin-1-ium [di-µ-bromido-caesium]] (I) Atomic displacement parameters (Å^2^)

**Table:**

	*U*^11^	*U*^22^	*U*^33^	*U*^12^	*U*^13^	*U*^23^
Cs1	0.01245 (18)	0.00867 (18)	0.01263 (18)	−0.00053 (6)	0.000	0.000
Br1	0.0165 (2)	0.0071 (2)	0.0255 (3)	0.00065 (10)	0.000	0.000
Br2	0.0141 (2)	0.0171 (2)	0.0185 (2)	0.000	0.000	−0.00791 (13)
C1	0.0259 (16)	0.0170 (17)	0.0187 (19)	−0.0048 (8)	−0.0058 (9)	0.0063 (10)
C2	0.0238 (15)	0.0173 (11)	0.0216 (12)	−0.0030 (10)	0.0029 (12)	−0.0062 (9)
N1	0.0218 (19)	0.0067 (14)	0.0367 (16)	0.0036 (12)	0.000	0.000
N2	0.0182 (16)	0.0104 (14)	0.0272 (14)	0.0037 (11)	0.000	0.000

### Poly[piperazin-1-ium [di-µ-bromido-caesium]] (I) Geometric parameters (Å, º)

**Table:**

Cs1—Br2^i^	3.5157 (5)	C1—H1A	0.9900
Cs1—Br2^ii^	3.5157 (4)	C1—H1B	0.9900
Cs1—Br2^iii^	3.5801 (5)	C2—N2	1.465 (3)
Cs1—Br2	3.5801 (5)	C2—H2A	0.9900
Cs1—Br1	3.6228 (6)	C2—H2B	0.9900
Cs1—Br1^i^	3.6392 (7)	N1—H1N	0.92 (4)
C1—N1	1.488 (4)	N1—H2N	0.84 (4)
C1—C2	1.510 (3)	N2—H3N	0.95 (4)
			
Br2^i^—Cs1—Br2^ii^	81.534 (14)	N1—C1—C2	110.2 (2)
Br2^i^—Cs1—Br2^iii^	132.072 (12)	N1—C1—H1A	109.6
Br2^ii^—Cs1—Br2^iii^	80.366 (13)	C2—C1—H1A	109.6
Br2^i^—Cs1—Br2	80.366 (12)	N1—C1—H1B	109.6
Br2^ii^—Cs1—Br2	132.072 (12)	C2—C1—H1B	109.6
Br2^iii^—Cs1—Br2	79.768 (14)	H1A—C1—H1B	108.1
Br2^i^—Cs1—Br1	135.970 (7)	N2—C2—C1	110.0 (2)
Br2^ii^—Cs1—Br1	135.970 (7)	N2—C2—H2A	109.7
Br2^iii^—Cs1—Br1	84.402 (12)	C1—C2—H2A	109.7
Br2—Cs1—Br1	84.401 (13)	N2—C2—H2B	109.7
Br2^i^—Cs1—Br1^i^	85.085 (12)	C1—C2—H2B	109.7
Br2^ii^—Cs1—Br1^i^	85.085 (13)	H2A—C2—H2B	108.2
Br2^iii^—Cs1—Br1^i^	136.466 (7)	C1—N1—C1^iii^	110.9 (3)
Br2—Cs1—Br1^i^	136.466 (7)	C1—N1—H1N	111.5 (11)
Br1—Cs1—Br1^i^	78.173 (18)	C1^iii^—N1—H1N	111.5 (11)
Cs1—Br1—Cs1^iv^	78.173 (17)	C1—N1—H2N	110.7 (13)
Cs1^iv^—Br2—Cs1^v^	100.865 (16)	C1^iii^—N1—H2N	110.7 (13)
Cs1^iv^—Br2—Cs1^vi^	178.768 (8)	H1N—N1—H2N	101 (3)
Cs1^v^—Br2—Cs1^vi^	80.367 (13)	C2^iii^—N2—C2	110.5 (3)
Cs1^iv^—Br2—Cs1	80.366 (13)	C2^iii^—N2—H3N	111.4 (10)
Cs1^v^—Br2—Cs1	178.768 (8)	C2—N2—H3N	111.4 (10)
Cs1^vi^—Br2—Cs1	98.402 (15)		
			
N1—C1—C2—N2	57.7 (3)	C1—C2—N2—C2^iii^	−60.2 (3)
C2—C1—N1—C1^iii^	−55.9 (3)		

Symmetry codes: (i) *x*−1, *y*, *z*; (ii) *x*−1, *y*, −*z*+1/2; (iii) *x*, *y*, −*z*+1/2; (iv) *x*+1, *y*, *z*; (v) *x*+1, −*y*+1/2, −*z*; (vi) *x*, −*y*+1/2, −*z*.

### Poly[piperazin-1-ium [di-µ-bromido-caesium]] (I) Hydrogen-bond geometry (Å, º)

**Table:**

*D*—H···*A*	*D*—H	H···*A*	*D*···*A*	*D*—H···*A*
N1—H1*N*···N2^iv^	0.92 (4)	1.95 (4)	2.868 (4)	179 (3)
N1—H2*N*···Br1	0.84 (4)	2.45 (4)	3.284 (3)	174 (4)
N2—H3*N*···Br2^vii^	0.95 (4)	3.07 (3)	3.756 (2)	130 (1)
N2—H3*N*···Br2^viii^	0.95 (4)	3.07 (3)	3.756 (2)	130 (1)

Symmetry codes: (iv) *x*+1, *y*, *z*; (vii) −*x*, −*y*+1, *z*+1/2; (viii) −*x*, −*y*+1, −*z*.

### Poly[piperazin-1-ium [di-µ-bromido-rubidium]] (II) Crystal data

**Table:**

(C_4_H_11_N_2_)[RbBr_2_]	*D*_x_ = 2.378 Mg m^−^^3^
*M**_r_* = 332.44	Mo *K*α radiation, λ = 0.71073 Å
Orthorhombic, *P**b**c**m*	Cell parameters from 1939 reflections
*a* = 4.4675 (13) Å	θ = 2.9–27.5°
*b* = 23.036 (7) Å	µ = 13.87 mm^−^^1^
*c* = 9.021 (3) Å	*T* = 93 K
*V* = 928.4 (5) Å^3^	Rod, colourless
*Z* = 4	0.20 × 0.05 × 0.05 mm
*F*(000) = 624	

### Poly[piperazin-1-ium [di-µ-bromido-rubidium]] (II) Data collection

**Table:**

Rigaku Pilatus 200K CCD diffractometer	771 reflections with *I* > 2σ(*I*)
Radiation source: rotating anode	*R*_int_ = 0.086
ω scans	θ_max_ = 25.3°, θ_min_ = 2.9°
Absorption correction: multi-scan (CrystalClear; Rigaku, 2013)	*h* = −5→5
*T*_min_ = 0.597, *T*_max_ = 1.000	*k* = −27→25
11351 measured reflections	*l* = −10→10
908 independent reflections	

### Poly[piperazin-1-ium [di-µ-bromido-rubidium]] (II) Refinement

**Table:**

Refinement on *F*^2^	Primary atom site location: structure-invariant direct methods
Least-squares matrix: full	Hydrogen site location: mixed
*R*[*F*^2^ > 2σ(*F*^2^)] = 0.023	H-atom parameters constrained
*wR*(*F*^2^) = 0.057	*w* = 1/[σ^2^(*F*_o_^2^) + (0.036*P*)^2^] where *P* = (*F*_o_^2^ + 2*F*_c_^2^)/3
*S* = 0.94	(Δ/σ)_max_ < 0.001
908 reflections	Δρ_max_ = 0.74 e Å^−^^3^
48 parameters	Δρ_min_ = −0.47 e Å^−^^3^
0 restraints	

### Poly[piperazin-1-ium [di-µ-bromido-rubidium]] (II) Special details

**Table:**

Geometry. All esds (except the esd in the dihedral angle between two l.s. planes) are estimated using the full covariance matrix. The cell esds are taken into account individually in the estimation of esds in distances, angles and torsion angles; correlations between esds in cell parameters are only used when they are defined by crystal symmetry. An approximate (isotropic) treatment of cell esds is used for estimating esds involving l.s. planes.

### Poly[piperazin-1-ium [di-µ-bromido-rubidium]] (II) Fractional atomic coordinates and isotropic or equivalent isotropic displacement parameters (Å^2^)

**Table:**

	*x*	*y*	*z*	*U*_iso_*/*U*_eq_	
Rb1	0.02991 (9)	0.30763 (2)	0.250000	0.01411 (14)	
Br1	0.52893 (9)	0.42482 (2)	0.250000	0.01792 (15)	
Br2	0.53856 (10)	0.250000	0.000000	0.02184 (16)	
C1	0.0103 (9)	0.56211 (19)	0.3859 (5)	0.0433 (13)	
H1A	−0.150237	0.532584	0.391919	0.052*	
H1B	0.138441	0.558250	0.474894	0.052*	
C2	−0.1233 (9)	0.62095 (17)	0.3812 (4)	0.0371 (10)	
H2A	−0.247265	0.627233	0.470786	0.045*	
H2B	0.037661	0.650481	0.380468	0.045*	
N1	0.1926 (10)	0.55235 (19)	0.250000	0.0490 (17)	
H1N	0.264148	0.515363	0.250000	0.059*	
H2N	0.351831	0.577000	0.250001	0.059*	
N2	−0.3067 (9)	0.62729 (18)	0.250000	0.0358 (12)	
H3N	−0.409568	0.662828	0.250000	0.043*	

### Poly[piperazin-1-ium [di-µ-bromido-rubidium]] (II) Atomic displacement parameters (Å^2^)

**Table:**

	*U*^11^	*U*^22^	*U*^33^	*U*^12^	*U*^13^	*U*^23^
Rb1	0.0154 (2)	0.0163 (3)	0.0106 (2)	−0.00032 (16)	0.000	0.000
Br1	0.0178 (3)	0.0145 (3)	0.0215 (3)	0.00069 (17)	0.000	0.000
Br2	0.0161 (3)	0.0330 (3)	0.0165 (3)	0.000	0.000	−0.01151 (19)
C1	0.050 (3)	0.045 (3)	0.035 (2)	−0.027 (2)	−0.031 (2)	0.024 (2)
C2	0.047 (2)	0.038 (2)	0.027 (2)	−0.024 (2)	0.020 (2)	−0.0183 (18)
N1	0.018 (2)	0.010 (2)	0.118 (6)	0.0018 (18)	0.000	0.000
N2	0.020 (2)	0.018 (2)	0.070 (4)	0.0028 (18)	0.000	0.000

### Poly[piperazin-1-ium [di-µ-bromido-rubidium]] (II) Geometric parameters (Å, º)

**Table:**

Rb1—Br2^i^	3.4157 (8)	C1—H1A	0.9900
Rb1—Br2^ii^	3.4157 (8)	C1—H1B	0.9900
Rb1—Br2	3.4659 (8)	C2—N2	1.447 (5)
Rb1—Br2^iii^	3.4659 (8)	C2—H2A	0.9900
Rb1—Br1	3.5013 (9)	C2—H2B	0.9900
Rb1—Br1^i^	3.5068 (9)	N1—H1N	0.9100
C1—C2	1.482 (6)	N1—H2N	0.9100
C1—N1	1.489 (5)	N2—H3N	0.9389
			
Br2^i^—Rb1—Br2^ii^	82.64 (3)	C2—C1—N1	109.6 (3)
Br2^i^—Rb1—Br2	80.96 (2)	C2—C1—H1A	109.8
Br2^ii^—Rb1—Br2	134.60 (2)	N1—C1—H1A	109.8
Br2^i^—Rb1—Br2^iii^	134.60 (2)	C2—C1—H1B	109.8
Br2^ii^—Rb1—Br2^iii^	80.96 (2)	N1—C1—H1B	109.8
Br2—Rb1—Br2^iii^	81.19 (3)	H1A—C1—H1B	108.2
Br2^i^—Rb1—Br1	135.144 (12)	N2—C2—C1	110.1 (3)
Br2^ii^—Rb1—Br1	135.143 (12)	N2—C2—H2A	109.6
Br2—Rb1—Br1	82.98 (2)	C1—C2—H2A	109.6
Br2^iii^—Rb1—Br1	82.984 (19)	N2—C2—H2B	109.6
Br2^i^—Rb1—Br1^i^	83.63 (2)	C1—C2—H2B	109.6
Br2^ii^—Rb1—Br1^i^	83.63 (2)	H2A—C2—H2B	108.2
Br2—Rb1—Br1^i^	135.505 (12)	C1—N1—C1^iii^	110.8 (4)
Br2^iii^—Rb1—Br1^i^	135.505 (13)	C1—N1—H1N	109.5
Br1—Rb1—Br1^i^	79.21 (3)	C1^iii^—N1—H1N	109.5
Rb1—Br1—Rb1^iv^	79.21 (3)	C1—N1—H2N	109.5
Rb1^iv^—Br2—Rb1^v^	100.02 (3)	C1^iii^—N1—H2N	109.5
Rb1^iv^—Br2—Rb1^vi^	179.021 (14)	H1N—N1—H2N	108.1
Rb1^v^—Br2—Rb1^vi^	80.96 (2)	C2—N2—C2^iii^	109.8 (4)
Rb1^iv^—Br2—Rb1	80.96 (2)	C2—N2—H3N	111.4
Rb1^v^—Br2—Rb1	179.021 (14)	C2^iii^—N2—H3N	111.4
Rb1^vi^—Br2—Rb1	98.06 (3)		
			
N1—C1—C2—N2	58.4 (4)	C1—C2—N2—C2^iii^	−62.1 (5)
C2—C1—N1—C1^iii^	−55.3 (5)		

Symmetry codes: (i) *x*−1, *y*, *z*; (ii) *x*−1, *y*, −*z*+1/2; (iii) *x*, *y*, −*z*+1/2; (iv) *x*+1, *y*, *z*; (v) *x*+1, −*y*+1/2, −*z*; (vi) *x*, −*y*+1/2, −*z*.

### Poly[piperazin-1-ium [di-µ-bromido-rubidium]] (II) Hydrogen-bond geometry (Å, º)

**Table:**

*D*—H···*A*	*D*—H	H···*A*	*D*···*A*	*D*—H···*A*
N1—H2*N*···N2^iv^	0.91	1.92	2.825 (6)	179
N1—H1*N*···Br1	0.91	2.40	3.300 (4)	171
N2—H3*N*···Br2^vii^	0.94	3.07	3.762 (3)	131
N2—H3*N*···Br2^viii^	0.94	3.07	3.762 (3)	131

Symmetry codes: (iv) *x*+1, *y*, *z*; (vii) −*x*, −*y*+1, *z*+1/2; (viii) −*x*, −*y*+1, −*z*.
